# What is Known About Critical Congenital Heart Disease Diagnosis and Management Experiences from the Perspectives of Family and Healthcare Providers? A Systematic Integrative Literature Review

**DOI:** 10.1007/s00246-022-03006-8

**Published:** 2022-09-20

**Authors:** S. Watkins, O. Isichei, T. L. Gentles, R. Brown, T. Percival, L. Sadler, R. Gorinski, S. Crengle, E. Cloete, M. W. M. de Laat, F. H. Bloomfield, K. Ward

**Affiliations:** 1 Liggins Institute, The University of Auckland, Auckland, New Zealand; 2 Te Whatu Ora, Auckland, New Zealand; 3 National Hauora Coalition, Auckland, New Zealand; 4 Department of Paediatrics, The University of Auckland, Auckland, New Zealand; 5 Heart Kids New Zealand, Tamariki Manawa Maia, Auckland, New Zealand; 6 Ngāi Tahu Māori Health Research Unit, Division of Health Sciences, University of Otago, Dunedin, New Zealand; 7 Te Whatu Ora, Christchurch, New Zealand; 8 School of Nursing, The University of Auckland, Auckland, New Zealand

**Keywords:** Congenital heart disease, Diagnosis, Qualitative research, Healthcare delivery, Fetal medicine

## Abstract

The experience of diagnosis, decision-making and management in critical congenital heart disease is layered with complexity for both families and clinicians. We synthesise the current evidence regarding the family and healthcare provider experience of critical congenital heart disease diagnosis and management. A systematic integrative literature review was conducted by keyword search of online databases, MEDLINE (Ovid), PsycINFO, Cochrane, cumulative index to nursing and allied health literature (CINAHL Plus) and two journals, the Journal of Indigenous Research and Midwifery Journal from 1990. Inclusion and exclusion criteria were applied to search results with citation mining of final included papers to ensure completeness. Two researchers assessed study quality combining three tools. A third researcher reviewed papers where no consensus was reached. Data was coded and analysed in four phases resulting in final refined themes to summarise the findings. Of 1817 unique papers, 22 met the inclusion criteria. The overall quality of the included studies was generally good, apart from three of fair quality. There is little information on the experience of the healthcare provider. Thematic analysis identified three themes relating to the family experience: (1) The diagnosis and treatment of a critical congenital heart disease child significantly impacts parental health and wellbeing. (2) The way that healthcare and information is provided influences parental response and adaptation, and (3) parental responses and adaptation can be influenced by how and when support occurs. The experience of diagnosis and management of a critical congenital heart disease child is stressful and life-changing for families. Further research is needed into the experience of minority and socially deprived families, and of the healthcare provider, to inform potential interventions at the healthcare provider and institutional levels to improve family experience and support.

## Background

Antenatal care aims to provide positive pregnancy outcomes and experiences for women [1]. Antenatal ultrasound is a recommended aspect of care for screening of congenital abnormalities (inborn defects that affect a child’s physical structure or function) [1, 2]. Congenital anomalies are a significant cause of infant and child mortality and morbidity worldwide [2]. The most common fetal anomaly is congenital heart disease (CHD) [2]. Outcomes for CHD have improved with the modernisation of diagnostic and surgical methods [3]. Recent advancements have also improved the outcomes of the most severe CHD subgroup, Critical Congenital Heart Disease (CCHD). CCHD is a term for congenital cardiac diagnoses which require intervention in the neonatal period for survival [3, 4]. Infants within the CCHD group who function on a single ventricle, termed Hypoplastic Left Heart Syndrome (HLHS), have the highest risk of mortality and are among the most medically complicated and controversial to manage [4, 5].

The parental experience of decision-making following a diagnosis of a fetal abnormality is complex and challenging [6–9]. Scholars and clinicians acknowledge that receiving an unexpected diagnosis of congenital heart disease is significant, usually resulting in parental grief and adaptation [8–10]. For more than fifty years, investigators have endeavoured to understand the nuclear (couple and dependent children) and wider extended family experience when parents receive a diagnosis of a congenital cardiac anomaly [11]. The continuum of intense stress from the time of life-threatening (often fatal) diagnosis onwards leads to anxiety, depression and post-traumatic stress disorder, negatively affecting parenting practices and infant-parent bonding [12–19]. Despite increased knowledge about how mothers adapt following a fetal anomaly diagnosis, evidence about the whole (nuclear and wider) family experience of a life-threatening congenital cardiac diagnosis and treatment along the continuum of this distressing journey is not well understood [18–24].

The healthcare provider is integral to how the family experiences healthcare. Exploring healthcare provider experiences of diagnosing and managing CCHD infants could give insight into how values and perceptions may differ from patients during the decision-making process [25–29]. As the number of studies investigating the family experience of CCHD children increases, it would be expected that the physician stance and response increasingly would cater to the needs and lived experiences of families of CCHD infants. However, recent evidence suggests differing perspectives between parents and healthcare professionals caring for children with advanced heart disease in the hospital setting [30]. The gaps identified in patient-doctor communication related to key areas of cardiac disease status and, importantly, areas of prognosis [30]. Healthcare workers who care for infants with precarious survival may also feel emotionally burdened, with evidence supporting early input from palliative care facilitating coping for both family and provider [31].

Understanding provider and patient family experiences of the most life-threatening subset of the commonest fetal anomaly, CCHD, is, therefore, essential to inform best-practice healthcare delivery and facilitate coping. Thus, we aimed to synthesise current understanding about the family and healthcare provider experience of critical congenital heart disease diagnosis and management.

## Methods

We used a systematic integrative review approach to synthesise current evidence exploring the families’ (nuclear and wider family) and healthcare workers’ experiences of CCHD diagnosis and management. Integrative review uses a systematic approach to incorporate studies with diverse methodologies to draw upon a wide range of evidence [32, 33]. The resultant thematic synthesis of information aims to comprehensively deepen the knowledge and understanding about a particular healthcare phenomenon [32, 33].

### Search Strategy

The following search strategy used the online databases MEDLINE (Ovid), PsycINFO, Cochrane, cumulative index to nursing and allied health literature (CINAHL Plus) and two journals, the Journal of Indigenous Research and Midwifery Journal. These topic specific journals were searched to ensure a wide net was cast. Searches were carried out via title and keyword with search terms designed to capture variants of the review question with appropriate wildcards inserted to search for word truncations or variations in key terms (Table 1). Papers prior to 1990 were not searched to retain relevance to current systems and practice and ensure clinical applicability of search results.

**Table 1:** Search terms

‘Experience’	‘Healthcare provider’	‘Family’	‘Critical congenital heart disease’
Experience* OR Perception* OR Perspective OR Interview* OR View* OR Opinion* OR Thought* OR Qualitative	Health personnel OR Healthcare OR Professional OR Practitioner OR Physician* OR Doctor* OR Nurse* OR Midwife OR Specialist*	Famil* OR Parent* OR Whānau OR Patient	Critical OR severe OR life-threatening AND Abnormal* OR defect OR disease or malform* AND Heart OR cardiac AND Congenital OR Inborn OR Antenatal

^*^Alternate endings applied during search

Pre-determined inclusion and exclusion criteria were applied to screen for eligibility by title, abstract, and full text (Table 2). Screening and final cross-checking was carried out independently by SW and OI, with consensus on the final 22 included articles confirmed by KW following discussion where ambiguity existed. One first author was contacted to confirm inclusion criteria were met [34].

**Table 2:** Integrative review inclusion and exclusion criteria

Inclusion	Exclusion
Papers exploring healthcare provider and/or patient families’ experiences of critical congenital heart disease^ diagnosis and/or management	Papers not including any element of healthcare provider and/or patient families’ experiences of critical congenital heart disease^ diagnosis and/or management
Paper describes majority of participants (= > 50%) with a diagnosis of critical congenital heart disease	Single case study or minority of participants with critical congenital heart disease
English language version available	No English language version available
Any children/fetuses with comorbidities (including genetic diagnoses and syndromes)	Infants with acquired cardiac disease
Empirical study	Review articles
Papers from Jan 1990 to July 2021	Experiences of adult or adolescent patients with CCHD (> = 10 years of age)
Full text available	Papers prior to 1990
Peer-reviewed studies	Full text unavailable
	Dissertation studies not peer-reviewed

^Heart defects classified as critical congenital heart disease include: aortic interruption or atresia or hypoplasia; coarctation or hypoplasia or the aortic arch; D-transposition of the great arteries; double-outlet right ventricle; Ebstein’s anomaly; hypoplastic left heart syndrome; single ventricle; pulmonary atresia intact septum; tetralogy of Fallot; total anomalous pulmonary venous connection; tricuspid stenosis and atresia, and truncus arteriosus

### Data Extraction

Data extraction was completed using a pro forma, capturing relevant factors of the paper, study design and limitations [35]. Overall data relating to the family or provider experiences of CCHD diagnosis and treatment was noted.

### Data Evaluation

Study quality was independently assessed by SW and OI incorporating three critical appraisal approaches: the critical appraisal skills programme (CASP) [36]; the mixed methods appraisal tool (MMAT) [37] and a method developed by KW incorporating CASP with a quality framework suggested by Hawker and colleagues to systematically review studies from different paradigms [38]. Included papers were assessed by sections including methodological rigour, relevance to the research aim, appropriateness of the recruitment strategy, the standard of data collection and analysis, evidence of ethics and attention to potential biases such as whether there was reflexive analysis and study implications. The studies evaluated via these criteria were assessed by a categorical scoring system as good, fair, poor or very poor using a score out of 40. Quality was cross-checked between the two assessors and a consensus reached by a third assessor (KW) where ambiguity existed. The third assessor (KW) also cross checked 22% of the studies independently at random to ensure a rigorous and replicable assessment was reached.

### Data Synthesis

The thematic data analysis occurred primarily by SW in four phases (Table 3) [39]. Data were extracted and tabulated in the form of base codes followed by identification of common themes. Tabulation allowed themes to be clearly grouped and then refined. Themes were refined with input from OI and KW and finalised by consensus between KW, OI and SW.

**Table 3:** Phases of synthesis

Phase	Description
Phase 1: Immersion in the data	Re-reading included papers in detail and noting initial ideas
Phase 2: Initial data coding	Coding systematically across all included papers for common topics and features of patient or provider experiences
Phase 3: Identifying themes	Sorting codes into themes Double-checking that data fit with themes and with the review questions
Phase 4: Refining themes	Report writing to refine final themes

Adapted from Braun and Clarke, 2006 [39]

## Results

**Fig. 1:**
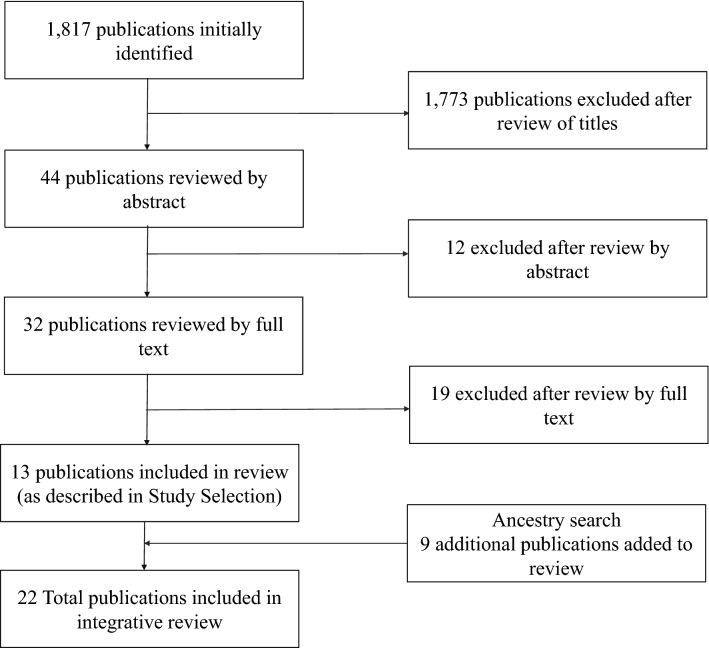
Flowchart showing literature selection

There were 1844 total studies identified during the initial electronic database search: 802 from Medline; 45 from PsycINFO; 685 from Cochrane, and 312 from CINAHL Plus. After exclusion of replicas, 1817 studies remained (Fig. 1).

The 22 included articles’ characteristics are summarised in Table 4. The majority of the 22 papers included in the review used qualitative methods. Three studies conducted focus group interviews [40–42], one study analysed journal entries [43], and the remainder conducted interviews. Two studies were mixed methods, including a quantitative arm with self-reported psychometric testing [44, 45]. One study triangulated their findings by assessing audio recordings of participant medical consultations [34]. Four studies had longitudinal data [42, 45–47], with two studies reporting on components of larger studies [42, 47]. Eighteen articles gathered cross-sectional information. Recruitment often occurred at large tertiary teaching hospitals and studies were conducted in Australia [44, 48], Canada [49, 50], Korea [51, 52], Norway [53], Sweden [54–56], Switzerland [57], the United Kingdom (UK) [34, 45, 58] and the United States of America (USA) [40–43, 46, 59, 60].

**Table 4:** List of included articles and their characteristics

Author, year	Setting	Study design	Sample (*n* =)	Ethnicity, marital status (when documented)	Sample (type)	Study quality
Gaskin, 2021 [45]	UK, specialist children cardiac centre	Prospective longitudinal mixed methods (interviews and self-report instruments)	*n* = 16 mothers and fathers	25% minority ethnicity, 91.6% living with partner	CCHD	Good
Hill, 2019 [42]	USA, major academic children’s hospital	Prospective longitudinal qualitative focus group interviews Secondary analysis of part of a larger study	*n* = 3 groups of mothers and fathers	33.3% minority ethnicity, 100% married	CCHD	Good
Cantwell-Bartl, 2013 [44]	Australia, tertiary hospital PICU	Cross-sectional mixed methods (retrospective narrative interviews and psychometric testing)	*n* = 29; 16 mothers and 13 fathers	17% English as a second language	HLHS	Good
Lisanti, 2017 [41]	USA, Children’s hospital of Philadelphia PICU	Cross-sectional qualitative focus group. Retrospective narrative interviews	*n* = 14 mothers in 3 focus groups	7% minority ethnicity, 93% married	CCHD	Good
Bertaud, 2020 [58]	UK, tertiary hospitals	Cross-sectional qualitative content analysis of retrospective narrative interviews	*n* = 8 mothers	Not documented	HLHS	Good
Ellinger, 2010 [49]	Canada, tertiary hospital	Cross-sectional qualitative retrospective narrative interviews	*n* = 25; 15 mothers and 10 fathers	Not documented	HLHS	Fair
Vandvik, 2000 [53]	Norway, National hospital	Cross-sectional qualitative retrospective narrative interviews	*n* = 20 mothers	80% remained in partnership with the father after birth	HLHS	Fair
Cantwell-Bartl, 2014 [48]	Australia, tertiary children’s hospital	Cross-sectional qualitative retrospective narrative interviews	*n* = 29; 16 mothers and 13 fathers	Not documented	HLHS	Good
Hwang, 2020 [52]	Korea, tertiary hospital	Cross-sectional qualitative retrospective narrative interviews	*n* = 9 fathers	Not documented	CCHD	Good
Harvey, 2013 [43]	USA, tertiary children’s hospital	Cross-sectional qualitative retrospective journal entry	*n* = 8 mothers	37.5% minority ethnicity, 75% married, 25% divorced	CHD	Good
Sood, 2018 [59]	USA, tertiary children’s hospital	Cross-sectional qualitative interviews	*n* = 34; 20 mothers and 14 fathers	53% minority ethnicity, 85% non-single parents	CHD	Good
Kim, 2017 [51]	Korea, tertiary hospital	Prospective cross-sectional qualitative interviews	*n* = 6 fathers	Not documented	CHD	Good
Harris, 2020 [46]	USA, tertiary children’s hospital	Prospective longitudinal qualitative interviews	*n* = 27; 16 mothers, 8 fathers and 3 support people	26% minority ethnicity, 18% English as a second language	CCHD	Good
Carlsson, 2016 [54]	Sweden, two tertiary referral hospitals	Prospective cross-sectional content analysis of qualitative interviews	*n* = 26; 14 mothers and 12 fathers (11 continued pregnancy and 15 terminated)	100% Swedish	CHD	Good
Carlsson, 2016 [55]	Sweden, tertiary referral hospital	Prospective cross-sectional qualitative content analysis of interviews	*n* = 9; 5 mothers and 4 fathers	100% minority ethnicity	CHD	Good
Lotto, 2018 [34]	UK, four fetal medicine sites, two tertiary referral hospitals	Prospective cross-sectional qualitative interviews and audio-recording of consults	*n* = 38; 20 mothers and 18 fathers	Not documented	Severe fetal anomalies	Fair
Woolf-King, 2018 [60]	USA, tertiary children’s hospital	Cross-sectional qualitative retrospective narrative interviews	*n* = 25; 15 mothers and fathers, 10 health care providers	Not documented	CHD	Good
Clark, 1999 [47]	USA, tertiary academic hospital	Prospective longitudinal qualitative interviews Secondary analysis of part of a larger study	*n* = 8 fathers	87.5% married parents	CHD	Good
Rempel, 2013 [50]	Canada, tertiary cardiac hospital	Cross-sectional qualitative grounded theory interview study	*n* = 53; 15 mothers, 10 fathers and 28 grandparents 17 grandmothers and 11 grandfathers, 15 young children	0% minority ethnicity, 100% Canadian	HLHS	Good
Thomi, 2019 [57]	Switzerland, tertiary academic children’s hospital	Prospective cross-sectional qualitative interviews	*n* = 18; 9 mothers and 9 fathers	0% minority ethnicity, 100% Swiss/Other European	CCHD	Good
Leuthner, 2003 [40]	USA, tertiary cardiac hospital, and obstetric unit	Cross-sectional qualitative focus group interviews (retrospective narrative)	*N* = 16; 4 parent focus groups; 9 mothers and 7 fathers (1 termination)	0% minority ethnicity, 100% Caucasian	CHD	Good
Bratt, 2015 [56]	Sweden, tertiary fetal unit	Prospective cross-sectional qualitative interviews	*n* = 12; 6 mothers and 6 fathers	100% coupled parents, 0% single parents	CHD	Good

*USA* United States of America, *PICU* Paediatric Intensive Care Unit, *UK* United Kingdom, *CHD* Congenital Heart Disease, *CCHD* Critical Congenital Heart Disease, *HLHS* Hypoplastic left heart syndrome, Minority ethnicity = non-white and immigrants

The overall quality of the included studies was generally good, apart from three of fair quality (Table 4) [34, 49, 53]. Participants were primarily English-speaking, married and well-educated. Studies lacked representation of minority ethnic groups, non-English speakers, single parents, and lower socioeconomic groups. Ten studies were retrospective narratives limited by possible recall bias. One study explored provider viewpoints on CCHD management, specifically in relation to the addition of mental health support services [60]. Twelve studies targeted CCHD alone, with a sub-group only assessing HLHS [44, 48, 49, 53, 58], the remaining ten included all CHD types. Common among studies was their focus on a discrete aspect of the experience (for example, the father’s experience or critical care experience).

Studies included in this review investigated the experience of diagnosis and/or management of CCHD for families and/or providers (data extraction summarised in Table 5). The healthcare provider perception was sought in one study, however, no insight was given into the provider experience, only the healthcare provider perception of the family experience. [60]

**Table 5:** List of articles and their key outcomes and data relating to the review question

Author, year	Key outcomes	Key points related to review question
Gaskin, 2021 [45]	This study explored parental experiences transitioning from hospital to home with their infant after stage 1 cardiac surgery for complex CHD. Thematic analysis identified evidence of acute stress disorder or post-traumatic stress responses along with anxiety and fear of discharge. Family coping, including anxiety and depression scores, generally improved over time	Parental wellbeing was negatively affected by a CCHD diagnosis with an ongoing significant impact of post-traumatic stress disorder for some individuals. Family functioning was also impacted, affecting family experience, especially with adaptations required when being discharged. Parity, family dynamic, and socioeconomic status influenced transition and parental wellbeing. Practical and financial stressors were identified alongside parental self-care and physical needs
Hill, 2019 [42]	The focus of this study was parent perception of family centred care in the PICU. Thematic analysis revealed that the physical and cultural environment negatively influenced how families experienced their child’s admission to the PICU. The parents appreciated respect for their spiritual needs	Parents experienced stress and uncertainty within the PICU environment. The parents valued positive interactions with healthcare providers, especially clinicians’ clear communication. It was also important that health professionals conveyed details regarding the infant’s care plan using language the parents understood. Having a consistent health care provider was also of value to families
Cantwell-Bartl, 2013 [44]	To evaluate the psychosocial status of mothers and fathers of infants with HLHS while in a tertiary hospital’s PICU. Findings revealed multiple stressors that began from the infant’s diagnosis and endured throughout the management. Post-traumatic stress disorder or acute stress disorder occurred in the majority (83%). There was no gender difference in stress reactions. There were gender differences in both experiences and the methods that assisted and hindered their adaptation process	Parental fear of death and universal distress occurred regardless of diagnosis timing. Participants reported a feeling of loss and marginalisation of their parental role with a lack of infant bonding due to the medicalised PICU environment. The medical equipment used, and the physical state of their infant, also added to their traumatic experiences. Supportive staff or other resources assisted adaptation. Family life and functioning was disrupted with resultant physical responses such as exhaustion. There were stressors from fragile relationships with healthcare providers due to communication breakdowns from insensitive comments, poor interpersonal skills, inadequate information, and low empathy
Lisanti, 2017 [41]	Explored causative factors of maternal stress of infants with complex CHD in the PICU in a large children’s hospital in the northeast mid-Atlantic region. A parental stress model may be used to guide future practices and research. Results confirmed consistency of their parental stress model in the PICU and added an additional theme which was time points of additional stress	Parents of infants younger than 1 year had significantly higher stress than parents of older children. Financial factors were a major feature adding to the stress of families. The experience of extreme stress impacted some participants’ physical health with evidence of flashbacks and symptoms of PTSD. The study also showed differential gender experiences, such as the impact on their parental role. Unexpected medical deteriorations and other uncertain situations also added to parental stress
Bertaud, 2020 [58]	This study undertook semi-structured interviews of HLHS mothers to explore the role of antenatal counselling in how parents make decisions following an antenatal diagnosis of HLHS. The results reported feelings of maternal guilt and the burden of decision-making they felt	Mothers of infants with HLHS responded to the diagnosis with emotional distress. They valued clear communication from healthcare providers with unbiased/neutral positioning. The experience of receiving the diagnosis and resultant decision making was impacted by family ethics/values with a role of the extended family
Ellinger, 2010 [49]	Interview data provided an insight into how parents who choose surgical palliation came to their treatment decisions for their child with HLHS. The key themes observed were trusting modern medicine, wanting to act, feeling like there was no other choice or it was God’s choice	Helpful nurses were key in supporting family decision making alongside their fundamental beliefs and values. The pressure of time constraints and the impact of faith were reported by participants
Vandvik, 2000 [53]	Mothers of HLHS infants were interviewed on how they experienced the decision-making process. Three main factors influenced how parents came to choose comfort care over surgery: more educated; a better childhood environment, and involvement in healthcare services	Mothers experience chaos and shock after receiving a diagnosis of HLHS, with compounded stress from the life-or-death decision they make for their infant. Parental perception of a healthcare provider’s favoured decision can impact the parent experience. The lack of knowledge, short time-frame and emotional distress from diagnosis shock might impact HLHS families’ decisions
Cantwell-Bartl, 2014 [48]	Semi-structured face-to-face interviews explored the psychosocial status of mothers and fathers regarding their response to their infant’s diagnosis of HLHS. The devastation was significant and, universally, with 83% saying that the time during and after the HLHS diagnosis was the most challenging time of their lives. There was an experience of loss, and the pressure of decision making and the impact of healthcare provider communication	The shock and stress were intense, with some having physical symptoms from this. There was evidence of trauma from poor provider communication. There were positive impacts on coping and adaptation from kind and supportive healthcare workers and other families. Rurality had an added impact on family functioning due to the physical distance from the hospital. Adaptation was supported by self-care, religion/spirituality and physical contact with their infant promoting bonding. Interviews outline how parents remembered details about the diagnosis process up to 19 years later
Hwang, 2020 [52]	The fathers' experiences of children with severe congenital heart defects in Korea were studied. In-depth interviews were thematically analysed with three themes emerging: “heart-breaking suffering”; “being a father of a child with CHD”, and “self-control during a great struggle”	The paternal experience of having a child with severe CHD involved great suffering and concern. Culturally these fathers perceived their role as supporting their wife and family, with a focus on how to financially help during the time from diagnosis. Adaptation occurred with self-control, re-framing and focusing on what is important in life
Harvey, 2013 [43]	This study analysed journal entries of mothers’ experiences of infants undergoing complex heart surgery. Thematic analysis identified one major theme of mothering through it all and five additional themes: intense fluctuating emotions; navigating medical information and practices; managing the unknown; facing the fearful potential their baby may die, and finding meaning and spiritual connection	The mothers all had difficult experiences. The unknown was fear-inducing with the responsibility of decision-making an additional stressor. The way information was communicated by healthcare providers was important for parents when navigating the medical world. The uncertainty of their child’s future was a stressor, including the possibility of death. The adaptation experience was supported by family, friends, religion/spirituality, and support people in a similar situation. There was also fear around how family functioning will be impacted
Sood, 2018 [59]	The goal of the study was to examine how mothers and fathers experience the stress of caring for a young child with CHD and utilise supports. The family experience of having a child with CHD was demanding and stressful. There was a gender difference in stress-inducing factors and supports used. Gender differences also occurred in how parents adapt to difficult situations and experiences	Participants reported generalised stress, fear and being overwhelmed with a “rollercoaster” of emotions. There was a fear of the unknown. With mistrust occurring in some, stress can increase from poor communication from providers such as inadequate information or ineffective communication between units and other hospitals. Financial and parenting burdens add to stress for families, which impacts marital functioning. The support they seek out is different and ranged from peer support, financial and work support, and spiritual and religious support
Kim, 2017 [51]	Study of the experiences of fathers of a neonate with CHD in Korea. The study outlined that Korean fathers had unique cultural characteristics comparative to western society. Care could be tailored to specific gender and cultural needs	Shock and misunderstanding when receiving the diagnosis occurred, along with a feeling of despair and lack of control. The emotional response to the diagnosis also encompassed guilt and fear and the burden of holding emotions in for their spouse's benefit. The word choice in the diagnosis discussion was important, with ‘deformity’ as a descriptive term not being well received by a parent. The adaptation response was improved by feelings of gratitude and practically having a plan for work and family functioning
Harris, 2020 [46]	The authors explored parents’ prenatal experience of CHD. The central theme was uncertainty; for instance, in terms of logistics and overall prognosis. Clinicians can assist with the framing of uncertainty at different time points	Timely and clear communication of the CHD diagnosis and plan with appropriate non-verbal communication was an important element of CHD diagnosis and management experiences of parents. Worrying about the unknown and the financial impact was also expressed. Access to support, including support group information, was thought to be valuable in aiding adaptation in the parental journey of having a child with CHD
Carlsson, 2016 [54]	The study explored prenatal parental information needs when a CHD diagnosis is made in the context of limited termination availability in Sweden. Content analysis revealed information was communicated in a complex and overwhelming way. Repeated, objective, clear information in their first language supported by pictures and text was assistive. In contrast, information obtained from the internet was difficult for families to interpret due to the non-specific and variable quality of information available	There was distress, shock, fear, confusion at the time of diagnosis. The feeling of being unprepared and having unanswered questions added to stress and impacted decision-making. Information that is matched to parental needs is integral in how parents adapt and cope with CHD diagnosis. At times parents experienced “insufficient” communication from the care team. There were also unmet needs around information around termination
Carlsson, 2016 [55]	The study explored the preferences and experiences of Swedish immigrants receiving a prenatal diagnosis of CHD. It showed that delay in receiving information increased distress. Participants valued trustworthy information, peer support and respect for their religious position	The way information was delivered was adequate, but more information in the ‘mother tongue’ was requested. The parents reported distress, worry and exhaustion. Support in a culturally appropriate way may be of benefit
Lotto, 2018 [34]	To explore parental decision-making using a comparative-based approach following a diagnosis of a severe congenital anomaly. Parents found navigating the uncertainty and decision-making process difficult. The way uncertainty was managed by the healthcare team influenced coping. Information to assist parents with their decision making was part of negotiating uncertainty. The perceived stigma and impact of religious organisations on termination decisions affected one parent	The parents experienced uncertainty about the diagnosis and severity alongside its prognosis and the decision required for future management. There was a negative impact on wellbeing when long wait times occurred in information delivery. The trust in the healthcare provider and system influenced the way parents navigated and accepted their child’s diagnosis. Being adequately informed empowered families and was associated with confidence in decision-making. This was influenced by education level
Woolf-King, 2018 [60]	Explored the impact of parenting a child with CCHD, including the provider perception. High-stress levels were associated with three-time points: diagnosis; cardiac surgery, and PICU stays. Distance from the providing hospital influenced the way the diagnosis impacted the mental health of families, the relationship with the care team and the total parenting burden (i.e., how many other children are there at home). The healthcare providers were quoted five times: twice regarding their support for increased mental health assistance for families; twice regarding the obvious trauma families experience, and once regarding communication barriers	There was vivid recollection of the grief, distress, shock, and despair experienced following a CCHD diagnosis. The families appreciated respectful dialogue with providers and access to mental health and peer support. The wider family needs also required adapting, which were important to how families coped. Provider information reflected their interpretation of how families’ experienced diagnosis and management and cope overall. One quote discussed communication: “So yes, I definitely have had experiences where the families have heard different messages from different players and felt like they contradicted. Sometimes they don’t contradict it’s just an interpretation that they’re contradictory. And that can escalate anxieties horribly.”
Clark, 1999 [47]	Explored the paternal experience of having an infant diagnosed with severe CHD. The results suggested conflicting reactions with a rollercoaster of emotions from joy to loss of control and fear of death. The fathers experienced a struggle as they tried to appear strong while hiding their intense emotions during the experience	There are unique conflicts with coping with the serious illness of a child for fathers. There is a joy of becoming a father but also the associated sadness, uncertainty and loss with the challenge of adapting to the infant’s vulnerable and fragile state. There was a struggle for the father to remain strong (especially for the mother) with a loss of the sense of control of the situation, amplifying the father's experience
Rempel, 2013 [50]	Reports on the results of a grounded theory study exploring the process of parenting young children who have survived HLHS. The extreme pressure of the situation of having a child with HLHS was central to the results of the study with the adjustment to the inconceivable, infant-attachment, managing the unexpected and new challenges	The emotional response to diagnosis was fear of death, shock, and distress. The process of adaptation to the HLHS diagnosis was managing uncertainty and adjusting expectations. The infant bond was also central and assisted in coping. There was also the balancing act of maintaining family functioning overall. The family support network as a coping strategy was clearly outlined
Thomi, 2019 [57]	Parental experiences from diagnosis of CHD to the first discharge after heart surgery was explored using a constructivist paradigm with inductive thematic analysis. When the CHD diagnosis occurred, the parents experienced shock, fear, uncertainty, and sadness. There also were increased parental demands and hands‐on work. Coping was ill-affected by poor healthcare provider communication and resultant distrust	Families identified times of heightened stress, such as receiving the CHD diagnosis, surgery and their infant’s PICU admission. A fractured relationship with healthcare professionals influenced the parental experience. There was also a focus on how the family and friends are managed as the nuclear family adapts to coping with the CHD diagnosis. The importance of having hope, increased infant bonding, and allowing themselves to feel the emotions assisted coping
Leuthner, 2003 [40]	This study explored the parental impact of an abnormal fetal echocardiogram, including emotions, attitudes, and coping strategies. The mothers voiced intense emotions of grief, guilt, fear, sadness, and hopelessness. Coping was assisted by infant bonding and having a realistic outlook on the future. The fathers were more likely to experience emotions, such as anger and anxiety. Fathers coped by focusing on their spouses and remaining optimistic	There was an overwhelming feeling of fear and grief experienced by parents. The fetal diagnosis affected the parental relationship and view of themselves. Parents felt fear for the future (uncertainty) and loss of control (including decision making). There were differential emotional reactions to the diagnosis and coping strategies adopted by gender. Coping mechanisms included detaching or attaching to the infant, denial, pessimism or optimism, privacy or participation in decision making
Bratt, 2015 [56]	The study explored the parental experience of counselling after a prenatal diagnosis of CHD to identify their needs. Content analysis resulted in three themes. First, parents valued clear information with adjuncts, such as brochures or recommended websites, to assist in understanding. Secondly, continued support with ease of access to providers throughout pregnancy, such as a paediatric specialist nurse, was of value. Peer support with couples who had similar experiences, including via social media, was also important. Thirdly, practical family functioning supports, including economic was a prevalent topic	The parents described the experience as the most stressful one in their life. Information and support needs differed between families. Coping was assisted by maintaining perspective. Families appreciated a detailed explanation regarding the logistics of care and hospital processes

*PICU* Paediatric Intensive Care Unit, *CHD* Congenital Heart Disease, *CCHD* Critical Congenital Heart Disease, *HLHS* Hypoplastic left heart syndrome, *PTSD* Post-traumatic Stress Disorder

We found that experiencing an unexpected diagnosis of CCHD universally is devastating and shocking for families. Thematic analysis revealed three themes:

1. Experiencing the diagnosis and treatment of a CCHD child significantly impacts parental health and wellbeing.
2. Parental responses and adaptation can be influenced by how healthcare and information is communicated and provided, and
3. parental responses and adaptation can be influenced by how and when support occurs.

*Theme 1* Experiencing the diagnosis and treatment of a CCHD child significantly impacts parental health and wellbeing

Families of children diagnosed with CCHD were significantly impacted in multiple spheres, including physical and mental health, family functioning and relationships [35, 37–55] There was also significant evidence of asynchronous responses between mothers and fathers, indicating that they respond differently to experiencing a diagnosis of CCHD and the process that follows [40, 41, 48, 51, 52, 59]. Intense emotional responses were universal between parents, including grief, shock, distress, depression, and anger [35, 37–55] Fathers were more likely to become angry than mothers [40]. Displaying characteristics of post-traumatic stress were common, with trauma resulting from diagnosis, seeing their children unwell (particularly in the unfamiliar intensive care setting) and unexpected events [44, 45, 48, 60]. Further stressors included the loss of their parenting role, painful time-pressured choices and feeling helpless from the loss of control [41, 44, 48, 58]. Loss of control was also of particular concern to fathers of CCHD infants [51, 52]. Mothers often felt guilty that their child’s CCHD was their fault, with the majority feeling the decisions regarding the management of the child’s condition (the choice of termination, continuation of pregnancy and active treatment or palliation) was ultimately their responsibility [43, 51, 58].

Fear was also a dominant feeling among parents of children with a CCHD diagnosis [40, 43, 47, 50, 51, 54, 57]. This fear became overwhelming at times, particularly of their child dying [45, 48]. There was also fear for the future and overall functioning of their child [40, 43]. This overwhelming fear was often coupled with uncertainty [34, 40, 42, 43, 46, 47, 49, 57]. Uncertainty was commonplace in experiencing the journey of having a CCHD infant—this uncertainty coupled with fear led to significant anxiety [40, 45]. This anxiety, in addition to the intense grief response, often led to parents describing physical impacts on their health such as exhaustion, feeling faint and tired. Compacting these physical sensations was the lack of self-care for their everyday physiological needs such as eating regularly and obtaining effective sleep [41, 42, 44, 45, 48].

Relationships were subsequently affected from experiencing a CCHD diagnosis, particularly within the nuclear family unit. There was evidence on the impact of the diagnosis on marriage/partnerships (positive and negative) from the strain of the experience and pressure from decision making [49, 57, 59]. Other siblings of the CCHD infant were also affected, with parents becoming less present but still having family commitments and demands, which needed to be balanced with the urgent demands of the CCHD child [48, 49, 51, 56]. Nuclear family functioning was also impacted by the demands of informing family and friends, with support for this greatly appreciated [57].

*Theme 2* Parental responses and adaptation can be influenced by how healthcare and information is communicated and provided.

The healthcare provider team is central to parental experiences of the diagnosis and management of their CCHD infant [53]. The healthcare team's communication, interaction, and framing of medical information is critical to maintaining a trusting patient-provider relationship [44, 57, 60]. The relationship with the care team influences the family’s ability to cope and how they respond and experience the critical diagnosis of their child [44, 57, 60].

Clear and informative provider communication was paramount to the family experience [46, 54, 58]. Families often commented on the communication from the healthcare team, particularly the lead provider [41, 42, 44, 46, 54, 57–60]. Patient families appreciated timely information communicated in full and positioned impartially [46, 58]. The use of words without subjective negativity was valued, for example, ‘difference’ rather than ‘defect’ [51]. There appeared to be a lasting impact of negatively perceived or pessimistic counselling with families remembering words to describe their child’s diagnosis vividly many years after the fact [53, 58]. The information required by families needed to be matched to their needs to not be too overwhelming or uninformative, and vague [56]. The information was also appreciated when conveyed with respect, including respect for a parent’s religious/spiritual or belief system with empathetic non-verbal cues [54, 60]. When there was perceived disrespect or withholding of information, fragility in the patient-provider relationship occurred with distrust in individuals and the health system [57]. It was unclear whether initial distrust in the health system influenced this response.

Repeated discussions regarding the diagnosis and management of CCHD infants was important to parents [54]. This included drawn diagrams, written information (particularly in the native tongue) and recommended websites [34]. Families felt empowered to make more informed and confident decisions based on correct information from validated sources [34]. Further, clearly communicated logistics were helpful for families, such as what was required of them and how to balance other commitments [56]. For example, having open access to a specialised nurse was helpful during pregnancy after a prenatal diagnosis of CCHD occurred [56]. Consistency was also key to improved experiences. [42, 60] This included consistency of information delivered and the health care professionals providing the care [42, 60].

Additionally, the location in which information and care was delivered impacted the parental experience [42, 44]. The environment in the hospital setting (the ward and intensive care particularly) limited privacy and was overwhelming for families with unfamiliar medical equipment attached to their unwell infant [44]. Infant-parent bonding was also compromised due to the physical constraints of medicalisation [44]. Parents reported barriers in forming an intimate emotional bond with the CCHD infant secondary to their reduced parental role alongside reduced time and space to do so [40, 44, 48, 49, 57].

*Theme 3* Parental responses and adaptation can be influenced by how and when support occurs

After the life-defining moment of diagnosis of their child with CCHD occurs, parents undertake a journey of adaptation [43–47, 50–52] How and when support occurs can shape how parents respond and adapt to their infant’s CCHD diagnosis [34, 46, 54, 60]. There are critical time-points when parental stress peaks and support needs appear to intensify [34, 46, 54, 60]. These time-points were identified as the time of diagnosis, decision-making (including deciding to terminate), birth or termination, at the point of surgery, entering intensive care post-surgery and discharge home [34, 46, 54, 60].

The way parents make sense of the CCHD diagnosis differs due to multiple factors such as underlying belief systems, available support networks and economic positioning [43, 46, 59]. Family functions also appear to impact adaptation responses as more dependants or those who live rurally appear to be more severely affected [44]. Therefore, practical supports such as economic (particularly for fathers and their work commitments), caregiving and informing and preparing for the expected and unexpected was appreciated [51, 56]. Particularly valued by families was support in the form of adequate information, compassion, thoughtfulness, and adequately managing uncertainty [34, 46, 55]. Further information on where to access peer support from individuals who had been in similar circumstances was also largely beneficial. Peers’ stories, often obtained from social media, the internet, and blogs, were valued by parents as they portrayed CCHD from another perspective than that of the healthcare system [56, 59, 60].

## Discussion

This review has identified the available evidence on how the family of an infant with CCHD experiences the diagnosis and subsequent management (22 studies), and the provider experience of CCHD diagnosis and management (one study). [13, 61, 62] This one-sided narrative depicts a possible disconnect between what families are experiencing and what providers perceive in healthcare delivery. The repercussions of the current research not revealing both the provider and the family experiences is that how the healthcare providers and system is perceiving and responding to the family perspectives cannot currently be clearly understood.

This review has also highlighted that minority groups, immigrants and those in more deprived social circumstances are currently underrepresented in the available research, even though these groups experience racism, classism and resultant distress. [13, 61–64] The impact of this current unavailability of literature on the broad range of family experiences on critical congenital heart disease health care interactions is that important issues relating to these underrepresented groups remain concealed.

A recent literature review of 94 papers on families’ experiences of having a child with any CHD (not just critical types) supports the significant psychological effects expressed in this review, including stress, anxiety, and depression [65]. Intense grief reactions occur following the traumatic news of a life-limiting fetal anomaly regardless of the decision to continue the pregnancy or terminate [18]. Potential harm from a disconnect of beliefs and values between the provider and patient can occur [66]. Future improvements in care quality during a life-defining event may facilitate a less traumatic experience, further assisting parental adaptation and care engagement [67]. Parents with fewer resources for support psychosocially are more at risk of lower wellbeing over time [68]. Wider literature also supports our finding on gender differences in suffering experiences and subsequent coping methods, which is influenced by their role in family functioning, sociocultural expectations, and knowledge [69–71].

Care quality impacts the experiences of families with a CHD child, as found in one study included in this review [65]. Parental responses and adaptation can be influenced by the quality of healthcare information and the manner in which it is communicated and provided [72–74]. Early, honest, impartial communication is integral to establishing a trusting relationship [75, 76]. Differing levels of trust are associated with different patient preferences in decision making [77]. A complex shift in communication is required to ensure uncertainty in diagnosis and prognosis is discussed and managed appropriately [78, 79]. Intensive care is a particularly important and parental fear-inducing setting where communication preferences currently are not met adequately, particularly for minority families [80, 81].

Parental responses and adaptation can be influenced by how and when support occurs. Holistic support of families is required, including caring for the family’s financial needs. The cost of living with, or caring for, a child with CHD is high, with evidence of a high personal burden related to economic effects from high disruptions to daily living and high health care utilisation [82, 83]. A review of eight articles describing parental experiences with a child undergoing heart surgery reported a major theme as ‘balancing the parental role’ [84]. This is in keeping with our findings that supporting the parental role and family functioning is vital to how parents experience CCHD diagnosis and management. Family psychosocial coping varies over time making the timing of support crucial [85, 86]. Healthcare systems need targeted and timely interventions to accommodate this fluidity in family needs. Holistic, culturally appropriate support, including facilitating parent-infant bonding (particularly for Indigenous peoples), could be developed—particularly as it has been associated with reduced maternal anxiety and improved attachment in CCHD [87, 88].

Evidence about provider perspectives on caring for and managing CCHD is lacking. The sparse data drawn from this review included data from health care providers who manage HLHS but without a specific focus on their experiences [5, 89]. Although the goodwill of health care providers is clear to families, their insight is lacking in some areas [25, 28, 90, 91]. There is an accordance of opinions of health care providers and parents regarding a child’s perceived quality of life in CHD but further insight into why consultations can appear ad hoc is required [25, 28, 90, 91]. This review is limited by search terms covering the umbrella term CCHD; therefore, some available evidence may have been missed by not searching each individual diagnosis. Similarly, some non-English speaking participant groups may not be captured in this review by the use of the English language inclusion criteria.

### Future Research Recommendations

An important focus for future inquiry is minority ethnic groups, non-English speaking, single parents, and low socioeconomic groups’ perspectives of CCHD diagnosis and management. Additional prospective longitudinal data on experiences of the pathway from prenatal diagnosis through to early childhood for parents with a CCHD child are needed. Integral to understanding this topic is understanding provider perspectives and experiences of CCHD care across the continuum (from diagnosis, counselling, and subsequent management). Broader research strategies could inform quality improvement so healthcare systems can function optimally, meet the needs of patients effectively and minimise potential harm. With future research aimed at understanding the full spectrum of family information and support needs, steps can be taken in maximising the effectiveness of interventions in these areas [92, 93]. Timely studies are also required due to the evolution of maternal–fetal surgery potentially contributing an added layer of management options for CCHD in the future [92, 93].

## Conclusion

The experience of diagnosis and management of a CCHD child is stressful and life changing for families. Opportunities for interventions at the healthcare provider and institutional levels are available to enhance healthcare quality. Focusing future prospective, longitudinal research on diverse family and family experiences of CCHD could inform best-practice healthcare delivery and facilitate coping for all.
